# Differential diagnosis of laryngeal spindle cell carcinoma and inflammatory myofibroblastic tumor – report of two cases with similar morphology

**DOI:** 10.1186/1746-1596-2-1

**Published:** 2007-01-09

**Authors:** Hans-Ullrich Völker, Matthias Scheich, Sylvia Höller, Philipp Ströbel, Rudolf Hagen, Hans Konrad Müller-Hermelink, Matthias Eck

**Affiliations:** 1 Institute of Pathology, University, Josef-Schneider-Str.2, 97080 Würzburg, Germany; 2 Department of Otorhinolaryngology, University, Josef-Schneider-Str.2, 97080 Würzburg, Germany

## Abstract

**Background:**

Spindle cell tumors of the larynx are rare. In some cases, the dignity is difficult to determine. We report two cases of laryngeal spindle cell tumors.

**Case presentation:**

Case 1 is a spindle cell carcinoma (SPC) in a 55 year-old male patient and case 2 an inflammatory myofibroblastic tumor (IMT) in a 34 year-old female patient. A comprehensive morphological and immunohistochemical analysis was done. Both tumors arose at the vocal folds. Magnified laryngoscopy showed polypoid tumors. After resection, conventional histological investigation revealed spindle cell lesions with similar morphology. We found ulceration, mild atypia, and myxoid stroma. Before immunohistochemistry, the dignity was uncertain. Immunohistochemical investigations led to diagnosis of two distinct tumors with different biological behaviour. Both expressed vimentin. Furthermore, the SPC was positive for pan-cytokeratin AE1/3, CK5/6, and smooth-muscle actin, whereas the IMT reacted with antibodies against ALK-1, and EMA. The proliferation (Ki67) was up to 80% in SPC and 10% in IMT. Other stainings with antibodies against p53, p21, Cyclin D1, or Rb did not result in additional information. After resection, the patient with SPC is free of disease for seven months. The IMT recurred three months after first surgery, but no relapses were found eight months after resurgery.

**Conclusion:**

Differential diagnosis can be difficult without immunohistochemistry. Therefore, a comprehensive morphological and immunohistochemical analysis is necessary, but markers of cell cycle (apart from the assessment of proliferation) do not help.

## Background

The most common type of malignant laryngeal tumors is the classical squamous cell carcinoma (SCC). Benign tumors of the larnyx are divided in two groups: mesenchymal and epithelial lesions. The latter harbours particular papillomas, whereas simple vocal cord polyps, Reinke edema, or e.g. leiomyomas have a mesenchymal origin. A further group are tumorous inflammatory lesions, such as granulomatous polyps. Most of these different entities show a characteristic histomorphology, so that the diagnosis might be unproblematic for the histopathologist.

Spindle cell lesions of the larynx are rare (1.3%) [1]. Such tumors usually require immunohistochemical investigations for detailed histopathological specification. In some cases, the dignity is difficult to determine.

We demonstrate a spindle cell carcinoma (SPC) and an inflammatory myofibroblastic tumor (IMT), two laryngeal spindle cell tumors with complete different dignity, and discuss the differential diagnosis focusing on the immunohistochemical results.

## Case presentation

### Clinical data

#### Case one

A 55 year-old male patient with relapsing dyspnoe and five pneumonias within the last four years was referred to our ENT hospital with progressive dyspnoe and dysphonia for five months. The patient was a smoker with 30 pack years, no alcohol abuse. He did not show any other systemic symptoms. Flexible transnasal laryngoscopy showed a laryngeal mass without visible glottis. Subsequently, microlaryngoscopy with laser resection of the ulcerated tumor (diameter 3 cm) was performed. The tumor originated from the right vocal fold. Histologically, a spindle cell carcinoma (SPC) was diagnosed. In a second surgery no remnants were found. The cervical lymph nodes were unsuspicious in ultrasound and computertomographic investigation. Therefore, neck dissection was not carried out. The patient is free of disease seven months after surgery.

#### Case two

A 34 year-old female patient with increasing dysphonia for one month was referred to our ENT hospital. She had neither a history of smoking nor alcohol abuse. Magnified laryngoscopy showed a polyp (0.8 cm) of the right vocal fold. Examination was followed by microlaryngoscopy with macroscopically complete resection. In a postoperative control ten days after surgery, a small nodule was found and suspected as a granulomatous polyp. Logopedic therapy led to a subjective voice improvement within the next three months. However, further magnified laryngoscopy showed an increasing size of the nodule. The following resection of a round tumor with 1.2 cm diameter was macroscopically and histologically complete. Eight months after surgery the patient is free of disease.

### Histopathological and immunohistochemical methods

After surgical resection, routinely processed paraffin blocks were cut at 2 μm and put on 3-aminopropyltriethoxysilane (APES) coated slides. Sections were first stained with hematoxylin-eosin (HE) and periodic acid Schiff (PAS) reaction.

Cuts for immunohistochemistry were air-dried over night, dewaxed, rehydrated in descending concentrations of ethanol before being heated for antigen unmasking in 10 mM citric acid (pH 5.5) for five minutes. After rinsing with distilled water, slides were washed in phosphate buffered saline (PBS). For staining, the Histostain-Plus bulk kit (Zymed) was used according to the manufacturer's protocol: 15 min blocking reagent, primary antibody incubation for one hour, rinsing with PBS (pH 7.4), biotinylated secondary antibody incubation for 20 minutes, rinsing with PBS, streptavidin peroxidase 20 minutes, and rinsing with PBS. Staining was performed by adding 3,3'-diaminobenzidine (DAB, Sigma) with subsequent counterstaining using haemalaun. The antibodies, sources, and dilutions used are indicated in table 1. In a first series, standard stainings for presumed diagnoses were carried out, a second was added obtaining further information. After the final diagnosis, an EBER (EBV-encoded RNA) in situ hybridization of case two was performed (EBER probe Y-5200, PNA-ISH-Detection Kit K-5201, Dako).

**Table 1:** Immunohistochemistry

**Antibody**	**Expression in SPC**	**Expression in IMT**	**Source/Dilution**
First stainings			

Vimentin *	**++**	**++**	DAKO, mouse, 1:800
PanCytokeratin AE1/3 *	**+**	**NR**	DAKO, mouse, 1:100
EMA	**+**	**+ (weak only)**	LOXO, mouse, 1:20
ALK-1 *	**NR**	**+**	DAKO, mouse, 1:20
smooth muscle Actin	**++**	**NR**	Beckman Coulter, mouse, 1:20
Desmin	**NR**	**NR**	DAKO, mouse, 1:400
S100	**NR**	**NR**	DAKO, rabbit, 1:2000
CD34	**NR**	**NR**	DAKO, mouse, 1:100
CD117	**NR**	**NR**	DAKO, mouse, 1:100
Ki67 *	**++ 60–80%**	**+ 5–10%**	DAKO, mouse, 1:200

Additional stainings			

PanCytokeratin KL1 *	**+**	**NR**	Beckman Coulter, mouse, 1:40
PanCytokeratin MNF116	**NR (but epithelium +)**	**NR**	DAKO, mouse, 1:50
Cytokeratin 5/6	**+ (weak only)**	**NR**	DAKO, mouse, 1:50
Cytokeratin 7	**NR**	**NR**	DAKO, mouse, 1:20
CD68	**NR**	**NR**	Kiel, ascites, 1:20000
CD30	**NR**	**NR**	DAKO, mouse, 1:5
CD56	**NR**	**NR**	DAKO, mouse, 1:10
Her2Neu	**NR**	**NR**	DAKO, rabbit, 1:100
Estrogen receptor	**NR**	**NR**	DAKO, mouse, 1:10
Progesteron receptor	**NR**	**NR**	DAKO, mouse, 1:10
p53	**+**	**+**	DAKO, mouse, 1:20
p63 *	**++**	**NR**	Neomarkers, mouse, 1:200
p21	**++**	**++**	Dianova, rat, 1:20
Cyclin D1	**+**	**+**	LOXO, mouse, 1:20
Bcl-2	**+ (weak cytoplasmatic)**	**++**	DAKO, mouse, 1:400
Rb	**+**	**++**	Neomarkers, mouse 1:50
HHV 8	**NR**	**NR**	Tebu, rat, 1:200
HPV	**NR**	**NR**	Virofem, mouse conc.

Used antibodies with expression pattern in spindle cell carcinoma (SPC) and inflammatory myofibroblastic tumor (IMT) as well as source and dilution. Staining started with antibodies in the upper part, followed by additional stainings, listed below. strong positive reaction (++), positive reaction (+), no reaction (NR), recommended for differential diagnosis (*).

All stainings are used in routine investigations and the antibodies were tested in suitable positive controls.

## Results

### Case one

**Figure 1:**
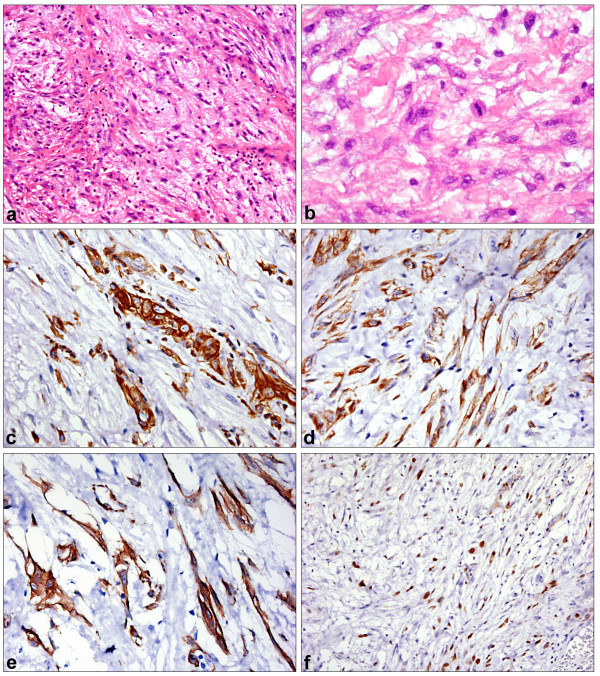
**Spindle cell carcinoma**. 1a: Spindle cell lesion with myxoid stroma (HE ×200). 1b: Mild to moderate atypia in tumor cells with rare mitoses (HE ×400). 1c: Vimentin immunoperoxidase – strong expression in tumor cells (×400). 1d: Pan-cytokeratin AE1/3 immunoperoxidase (×400). 1e: smooth muscle actin immunoperoxidase (×400). 1f: Proliferation (Ki67) up to 80% (immunoperoxidase ×200).

In HE staining, an ulcerated spindle cell tumor with widely myxoid stroma changes was found. Tumor cells were arranged in an irregular pattern with different cell density (figure 1a). No PAS positivity was found, necrosis was absent. Spindle cells showed mild to moderate atypia with prominent nucleoli. Ten high power fields (HPF) contained a maximum of one mitosis (figure 1b), and no atypical mitoses were found. Focally, the tumor was scantily infiltrated by neutrophil granulocytes. The surface epithelium was only detectable on a small focus, herein no atypia was found in the surface epithelium. The results of immunohistochemical investigations are indicated in table 1. The tumor was strongly positive for vimentin (figure 1c) and different cytokeratins including the squamous differentiation marker CK5/6 (figure 1d). Only pan-cytokeratin MNF116 was negative in the tumor cells, but reacted with the surface epithelium. Smooth-muscle (sm)-actin (figure 1e) was coexpressed. The proliferation (determined by Ki67 staining) was up to 80 % (figure 1f). Due to these features a *spindle cell carcinoma *was diagnosed. Together with the clinical information, the tumor stage was pT2, cN0, cM0, and (after resurgery) R0.

### Case two

**Figure 2:**
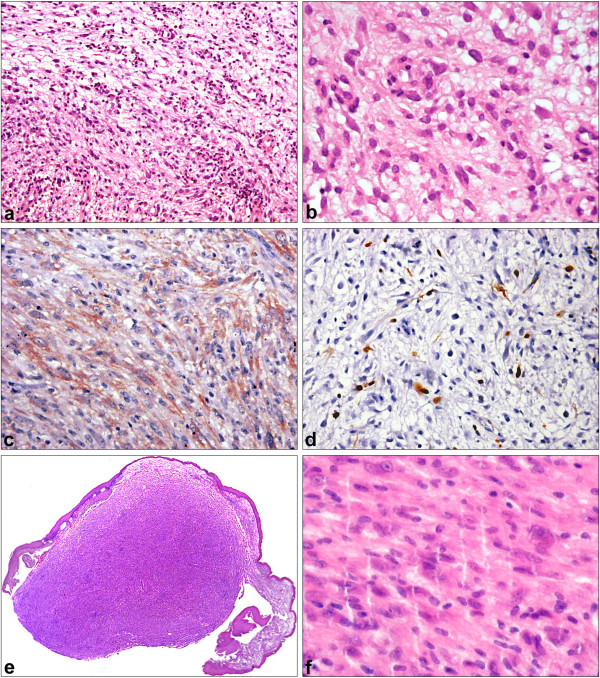
**Inflammatory myofibroblastic tumor**. 2a: Spindle cell lesion with more regular pattern than in 1a, myxoid degeneration, vessels similar to granulation tissue, infiltration with inflammatory cells (HE ×200). 2b: Lack of atypia in tumor cells, resembling regular myofibroblasts (HE ×400). 2c: Diagnostic expression of ALK-1 immunoperoxidase (×400). 2d: Low proliferation (Ki67), less 10% (immunoperoxidase ×400). 2e: Relapse tumor, sharply confined surfaced with intact epithelium (HE ×40). 2f: Higher cellularity, no myxoid changes, less inflammatory cells, but mild atypia in the relapse (HE ×400).

This lesion showed an organized growth pattern of spindle cells. Most of the tumor stroma was myxoid (figure 2a). The tumor had regular small blood vessels and capillaries similar to granulation tissue. A scanty infiltration with inflammatory cells (dominated by lymphocytes) was found. Plasma cells were rare, neutrophils were absent. No necrosis was found. Tumor cells showed only mild atypia (figure 2b). The spindle shaped nuclei presented mostly blunt ends. Chromatin was evenly distributed, nucleoli were not visible. We did not identify mitoses. As proof of our final diagnosis of an *inflammatory myofibroblastic tumor*, immunohistochemistry revealed an expression of ALK-1 (figure 2c). Corresponding to the benign character, the proliferation (Ki67) was less than 10 % (figure 2d). Table 1 shows other results of immunohistochemical investigations. The EBER in situ hybridization did not reveal an infection with Ebstein-Bar-Virus (EBV). The relapse was a sharply confined nodule below an intact surface epithelium (figure 2e). The cell density was higher and myxoid changes were absent (figure 2f). No mitoses were found, but mild atypia. The number of inflammatory cells was reduced. Immunohistochemical pattern was identical to the primary lesion.

Apart from the diagnostic areas shown, both cases contained distended spindle cell components with a high degree of overlapping histomorphology in conventional HE stain.

## Conclusion

We demonstrated two cases of laryngeal spindle cell tumors with complete different dignity. The first case was a spindle cell carcinoma (SPC), the second an inflammatory myofibroblastic tumor (IMT). Because of similar morphology, only comprehensive immunohistochemical investigations allowed the correct final diagnoses and the aligned assessment of biological behaviour.

According to the recent WHO definition, laryngeal SPCs represent biphasic tumors, composed of a squamous cell carcinoma (SCC), either in situ or invasive, and a malignant spindle cell component with mesenchymal appearance [2], but of an evident monoclonal epithelial origin [3]. Obsolete synonyms are sarcomatoid carcinoma, carcinosarcoma, collision tumor, or pseudosarcoma. Males in the 6^th ^and 7^th ^decade are predisposed (reported male to female ratio is 10:1) [4,5]. The vocal fold is the most common site of involvement [5]. Risk factors correspond to common SCC: cigarette smoking and alcohol abuse [4]. Some SPCs seem to develop after radiation [5,6], but a strong evidence of this hypothesis is missing. The prognosis of SPCs is discussed controversially. One quarter metastasizes in regional lymph nodes, distant metastases occur in 5–15% [5]. These often contain usual SCC with or without spindle cell component. Rarely, only the spindle cell component occurs [6]. Five year survival is reported to be 65–95% [1,5]. Low tumor stage and absence of previous irradiation indicate a better prognosis. Besides, improved survival rates are associated with a low level of cytokeratin expression [6].

The differential diagnosis includes, apart from rare laryngeal sarcomas, reactive or benign spindle cell proliferations, such as nodular fasciitis, IMT, or low grade myofibroblastic sarcoma.

IMTs are neoplasms with borderline dignity [2]. They are composed of myofibroblastic cells and intermingled inflammatory cells, especially plasma cells [7]. Nowadays, obsolete synonyms are inflammatory pseudotumor, plasma cell granuloma, plasma cell pseudotumor, or pseudosarcomatous lesion/tumor [2]. The latter is similar to the synonymous "pseudosarcoma" for SPC and might lead to severe misunderstandings. The most common location is the lung, followed by soft tissue and viscera. IMT of the head and neck, especially of the larynx, are rare [7,8]. Coffin described 12 cases of head and neck IMTs among 84 extrapulmonary IMTs, and only three were located in the larynx [7]. 19 cases of laryngeal IMTs have been reported in the literature. All cases are listed in table 2. In distinction to IMTs occuring in other sites, predominantly in children or young adults, IMTs of the larynx affect adults with a median age of 57 years (2–74). The male to female ratio is 1.8:1. Only one case is reported in a child [9]. The prognosis is excellent. Metastases are possible, but were not decribed for IMTs of head and neck and did not occur in the reported cases [7]. The recurrence rate was 21%. Most relapses occurred within 12 months after initial surgery, perhaps due to incomplete resection in most instances. One problem for surgeons is the lack of a line of demarcation surrounding the lesion. Radical surgery is reserved for more aggressive cases [8-10]. However, a total laryngectomy was necessary only in one of all IMTs reported [8]. Some patients profit from corticosteroid [11] and nonsteroidal anti-inflammatory treatment[12].

**Table 2:** Synopsis of all 19 laryngeal inflammatory myofibroblastic tumors published in the English literature.

**Patient, Age**	**Symptoms**	**Site**	**CS**	**Course**	**Immunostaining**
M, 62 [20]	Hoarseness, dysphonia, systemic manifestations (fever and weight loss) for 3 months	True vocal cord	yes	FD 24 months	vim +, actin +, NR: desmin, CK, S100, Alk-1
M, 2 [9]	Snoring, apneic events	Aryepiglottic fold	no	R 7 months	*
F, 52 [11]	Voice change for 12 months	False cord	no	FD 4 years	vim +, sm-actin +, NR: S100, desmin
F, 49 [10]	Dyspnoea, stridor for 4 month, 15 month after road accident	Subglottis	no	Persistence for 1 months after surgery, resurgery with persistence for 12 months, 2^nd ^resurgery, FD 15 months after resurgery	sm-actin patchy +, NR: desmin, CK, EMA
M, 51 [32]	Hoarseness for 6 months	Vocal cord	*	FD 12 months	vim+, sm-actin+, NR: CK, desmin, S100, CD34
M, 74 [33]	Hoarseness, foreign body sensation, dyspnoe for several months	True vocal cord	*	FD 2 months, death on cardiac failure	vim +, actin +, NR: S100, desmin, CK
M, 57 [34]	Hoarseness, dyspnoe, disphonia for 5 months	Anterior commissur	*	R 2 months, after resurgery FD 12 months	vim +, actin +
M, 72 [35]	Dysphonia for 2 months	True vocal cord	*	FD 6 months	vim +, sm-actin +, CD68 focally +, NR: CK, S100, desmin, CD34, LMP
5 M, 3 F with age median of 59 (19–69) [8]	Hoarseness, stridor, dysphonia, foreign body sensations from 10 days up to 4 months	*	3+/8	7 cases FD, one case with 4 relapses in 24 months	All: vim +, sm-actin +, NR: CK, S100, desmin, myoglobin, CD34
3 further cases, [7]	*	*	*	2 cases FD, one R 12 months	*

Male (M), Female (F), Cigarette smoking (CS +), Free of disease (FD), Recurrence (R), Vimentin (vim), Cytokeratin (CK), positive (+), no reaction (NR), R * not specified or not reported.

The etiology of IMTs is unknown. The idea of a reactive lesion was refuted by genetic studies. The clonal origin is evident [13,14] and chromosomal abnormalities involving 2p23 [15,16], and fusion of the ALK gene with tropomyosin 3 (TPM3-ALK) or tropomyosin 4 (TPM4-ALK) is found in a subset [17]. Gene rearrangement and gene activation are restricted to the myofibroblastic component. Some authors assume an association with a trauma. Alaani et al. have documented a causal relation between local laryngeal trauma and IMT [10], Wenig et al. found an IMT after traumatic intubation [8]. Furthermore, immunosuppression, unspecific infections, infections of HHV8 [18] and EBV [19] were identified in IMT and an overexpression of IL-6 and cyclin D1 (confirmed by our investigations) have been reported [18]. In the case we have presented, we could not affirm any infectious genesis.

Clinically, IMT can mimic a neoplastic process. Patients suffer from hoarseness, dysphonia, or foreign body sensations in the troath. Constitutional or systemic signs (fever, weight loss, anaemia) are usually missing in extrapulmonary IMTs. Systemic alterations are only reported in one laryngeal IMT [20].

The diagnosis of IMTs can be difficult due to the wide morphological spectrum. Coffin et al. have described three morphologic patterns:

1. spindle cells in a myxoid background with a vascular and inflammatory component (nodular fasciitis like)

2. compact spindle cells in a solid confluent area or as irregular foci in areas of dense collagen (fibrous histiocytoma like)

3. collagen dense pattern similar to desmoid fibromatosis [7].

Our case of IMT first presented as variant one with relapse as variant two. Alaani et al. described in a relapsed IMT a progressive diminution of inflammatory cells, reduction of atypia and more collagenous, less myxoid stroma up to osteoblastic changes [10]. A transformation of an IMT to higher malignancy is possible, especially in cases with repeated recurrences.

According to Coffin et al., immunohistochemical investigations could reveal an expression of vimentin in 99%, sm-actin in 92%, focally desmin in 69%, cytokeratins in 36%, CD68 in 24%, and CD30 in 6% [7]. The reported laryngeal IMT expressed consistently vimentin and actin, but none of the other markers. Anaplastic lymphoma kinase (ALK) gene expression is detectable in IMTs, often in children and young adults under 40 [14-17,21]. Only two laryngeal IMTs (including our case) have a reported staining with antibodies against ALK-1. The importance of ALK expression regarding the prognosis is controversially discussed. Chun et al. found a better prognosis in cases with ALK expression [22], whereas Coffin et al. found a recurrence in 45% of ALK-positive and 20% of ALK-negative IMTs [16]. The malignant transformation seems independent of ALK expression [16].

The differential diagnosis of IMT comprises low grade myofibroblastic sarcomas as well as a long list of benign, reactive, or neoplastic spindle cell lesions, such as leiomyoma, solitary fibrous tumor, spindle cell carcinoma, nodular fasciitis, and peripheral nerve sheet tumor [20]. Morphology and immunohistochemical profile help to rule out these entities, whereby low grade myofibroblastic sarcoma can be difficult to distinguish due to the low or mild atypia and low proliferations index [23]. The best discrimination of this malignant neoplasm with favorable prognosis is the occurrence of local infiltrative growth [20,23].

As mentioned above, SPC is also an important differential diagnosis. But if the diagnosis cannot be made on the basis of conventional histomorphology, it becomes even more difficult. Typically, SPCs contain pleomorphic malignant spindle cells with mitoses (including atypical mitoses). Most of them are associated with epithelial dysplasia or common SCC [5]. Our case of SPC showed similarities with IMT in some areas. We found only mild atypia, hypocellular myxoid degeneration and no association with dysplasia of the surface epithelium. So we were not able to diagnose a SPC with HE staining alone. However, immunohistochemistry was evident for SPC/IMT (SPC: cytokeratin and vimentin positive, ALK-1 negative versus IMT: ALK-1 and vimentin positive, cytokeratins negative). Problems arise in an ambiguous staining pattern: SPCs express cytokeratins only in 40–85% and IMTs in up to 75% [5,7,8,24-26]. In our SPC, not all pan-cytokeratins (MNF 116) were expressed. In such cases, the p63 antibody is helpful [27]. An expression of sm-actin is reported in SPC and IMT [1,7,8], we found it only in SPC. In contrast to that, all laryngeal IMTs in literature expressed actin (table 2). An expression of ALK-1 is typical, but only one half of IMT are positive. And interestingly, positivity in patients over 40 years is less likely (whereas IMTs of the head and neck occurs in the 5^th^/6^th ^decade) [28,29]. Therefore, even comprehensive immunohistochemical analysis could fail in revealing the right diagnosis.

Hussong et al. described an overexpression of p53 in one of six IMT with recurrence and in one of two with malignant transformation, but in none without [30]. Even if Ledet et al. found p53 only in malignancies but not in IMTs [31]. But an expression of p53 seems to be not a sure sign of malignant behavior. Yamamoto et al. described p53 positivity in 6.7% of all IMTs [29], Brooks et al. detected it in an oral IMT without recurrence or malignancy [28]. However, the highest number of investigated IMTs is a study of 24 cases (including six recurrences and two malignant IMTs) [30]. Therefore, p53 seems to be not sufficient criterion for differential diagnosis. Bcl-2 expression is reported in 38% of IMT without relation to recurrence or malignant transformation [30]. Expression of cyclin D1 and Rb was described [18,28], the value of p21 is not assessed. The degree of cellularity, mitoses, and the extent of inflammatory cells showed no correlation to more aggressive behavior [30], but the presence of aneuploidy [16], atypia and ganglion-like cells do [30]. Reliable criteria with a high predictive value regarding the biological behavior of IMTs do not exist so far.

In summary, the differential diagnosis of SPC and IMT can be difficult, particularly in cases with uncommon immunohistochemical profile. Therefore, a comprehensive morphological and immunohistochemical analysis is necessary, but markers of cell cycle (apart from assessment of proliferation) do not help.

## Competing interests

The author(s) declare that they have no competing interests.

## Authors' contributions

HUV and MS drafted the manuscript, HUV, SH, PS, and ME evaluated the immunohistochemical stainings and confirmed the diagnoses, MS and RH compiled the clinical data, HKM and ME mainly contributed to the discussion. All authors read and approved the final manuscript.
